# Adolescents in acute mental health crisis—Pilot-evaluation of a low-threshold program for emotional stabilization

**DOI:** 10.3389/frcha.2023.1177342

**Published:** 2023-07-07

**Authors:** Andrea Dixius, Tanja Michael, Adriana Altpeter, René Ramos Garcia, Eva Möhler

**Affiliations:** ^1^ Saarland University, Saarbrucken, Germany; ^2^ SHG Clinic for Child and Adolescent Psychiatry, Saarbrucken, Germany; ^3^ Division of Clinical Psychology and Psychotherapy, Department of Psychology, Saarland University, Saarbrucken, Germany; ^4^ Saarland University Hospital, Homburg, Germany

**Keywords:** adolescents, emotion regulation, crisis, stress, mental health, stabilization, resilience

## Abstract

**Background:**

Referrals for child and adolescent acute psychiatric treatment have spiked in the last two years. To provide these adolescents with a fast-acting intervention, a novel treatment approach for acute emotional dysregulation was evaluated in this study.

**Methods:**

156 adolescents between the age of 13 and 18 years who were admitted to a psychiatric unit for acute emotional or behavioral dysregulation participated in a 5-week-group program (Stress-Arousal- Regulation-Treatment, START) which consisted of two sessions per week (60 min/session). Pre- and post intervention psychometric measures were derived for each participant applying the Self-Control Scale (SCS-13), the FEEL-KJ capturing adaptive and maladaptive emotion regulation strategies, the Perceived Stress Scale (PSS-10), as well as the Strengths and Difficulties Questionnaire (SDQ).

**Results:**

The mean score of maladaptive emotion regulation strategies of the FEEL-KJ significantly decreased post treatment (*d = *−0.24*, p = *.001), while there was an increase in adaptive emotion regulation strategies (*d *= 0.25, *p = *.001*).* The post- intervention-assessment revealed significantly lower scores in the PSS-10 (*d = *−0.34*, p < 0*.001), as well as SDQ mental health problems (*d *= −0.17*, p = *.018) and disorders (*d = *−0.15*, p *= .015). The Self-Control Score increased significantly after the *intervention (d = *0.21, *p = *.001*)*.

**Conclusions:**

In this large sample, the low-threshold intervention START significantly improved emotion regulation and self-control and reduced perceived stress as well as several scales of the strengths and difficulties questionnaire, pointing to a good feasibility and indicating efficiency to provide support to adolescents with acute mental health problems when applying this short-term treatment.

## 1. Introduction

Emotion regulation is central to well-being and mental health (1–3). Emotion regulation strategies are used to reinforce, maintain or reduce the extent of the affective experiences (3, 4). Feelings, perception, bodily reactions, and behavior are related. Maladaptive emotion regulation strategies influence the level of functioning of individuals applying them on a daily basis. Impairments in emotion regulation (5) which can be caused by experiencing stressful life events (6, 7) contribute strongly to the development and maintenance of mental illness and trauma-related symptoms (8–10). Dysfunctional strategies of emotion regulation such as self-harming behavior, impulsive behavior, or substance abuse are often put in service of managing tension states and dealing with perceived aversive emotions (13). The development of severe psychopathology in adolescents can be the result of adverse childhood experiences and chronic stress, resulting in emotion dysregulation (6, 14, 25). Early interventions have been found to be a counteracting factor to prevent chronification (15).

Recent studies report a continuous increase in mood dysregulation (7, 16) in adolescents. Emotion dysregulation is reported to be associated with exemplary maladaptive behaviors such as non-suicidal self-harming behavior, substance use, social withdrawal, and impulsive behaviors (12, 13, 17, 46). Maladaptive strategies are seen as dysfunctional attempts to regulate stress and perceived aversive emotions or emotions perceived as distressing, for example by executing self-harming and impulsive behavior (11, 12). Functional levels of personality cognition, behavior, social abilities and integration are restricted by emotion dysregulation and lead to a variety of problems in development (18, 19, 23, 26, 27).

Early stressful events and trauma have been described to impair neurobiological structures and functions related to emotion regulation, such as the orbitofrontal gyrus and frontolimbic connections (20, 21). Felliti and colleagues (6) were previously able to show that children and adolescents with invalidating experiences and traumatic and stressful life events have a higher risk of developing physical or psychological pathologies and addictions in later life in the Adverse Childhood Experiences Study. Schmid and colleagues (14) describe the accumulation of stress in the Trauma Developmental Heterotopy Framework. The influence of stress on emotion regulation seems to play a central role in linking stress and psychopathology.

Recent research in the field of emotional dysregulation (ED) has focused on identifying the underlying neural mechanisms and developing effective interventions. Studies have shown that certain regions of the brain, such as the amygdala and the prefrontal cortex, play a critical role in regulating emotions. Additionally, research has also highlighted the importance of the body's stress response system, specifically the HPA axis, in the development and maintenance of emotion dysregulation.

In general, early interventions seem to be of crucial importance in reducing mental health care costs and improving psychosocial functioning in children and adolescents (24, 28). Focusing on promising interventions, an area of recent research is the development of cognitive-behavioral therapies, such as dialectical behavior therapy (DBT), which has been shown to be effective in treating emotional dysregulation in individuals with conditions associated with emotion dysregulation such as borderline personality disorder (22). However, inclusion criteria for participation in a DBT-program are a commitment for participation to a 3-month treatment program, with considerable requirements regarding intelligence, attention span and motivation, which in an acute setting can only be fulfilled by a minority of patients. Therefore a program with less cognitive and motivational requirements was needed, thereby closing the gap for acutely referred patients that are not yet stable enough for more complex therapy programs, such as DBT-A.

The intervention START (Stress-Arousal-Regulation-Treatment) was designed to close this gap in mental health care for adolescents. Therefore, the goal of our study was to evaluate a novel DBT-based short-term treatment program (START) with regard to its ability to help adolescents referred to acute in-patient treatment with the symptoms described above, offering a low-threshold approach for acute stabilization.

The START program (29) is DBT-oriented and based on a dialectic attitude. In the present study, improvement of strengths and the reduction of psychological and social problems are of interest. More self-control could promote an important attribute for positive development. Self-control predicts physical health and counteracts severe impairments such as drug addiction, personal financial problems and criminal behavior by following a gradient of self-control. Moreover, self-control (34) could influence positive development, which is helpful for integration in social contexts. Furthermore, stress or perceived stress (PSS-10) affects an interdependent network of emotion regulation (34–36) and has an impact on mental and physical health. Therefore, a major target of this clinical evaluation was self-control, perceived stress and emotion regulation (5) as well as general psychopathology before and immediately after the short term intervention in patients referred to an acute psychiatric in-patient setting for symptoms of severe emotion dysregulation.

## 2. Material and methods

Between October 2021 and December 2022 162 patients were recruited to participate in the 5-week START study at an inpatient psychiatric unit with full emergency care obligation for a federal state in Germany. Drop-out occurred in 6 cases due to lack of motivation. The recruitment period led to 62 boys and 161 girls being admitted because of imminent danger for themselves or others to our acute ward. Apart from 3 girls and 2 boys with a first diagnosis of acute schizophrenic psychosis, 218 of these adolescents were approached and offered participation in the evaluational study of the START-program. 62 adolescents declined participation for lack of time or motivation. However, the gender ratio of participating adolescents is representative for the entire sample. The grand total of all adolescents referred to acute treatment consisted of 27.8% male adolescents, which is similar to the ratio found in our final sample.

### 2.1. Sample

The final sample (*n* = 156) included for analysis consisted of 36 male and 120 female patients aged between 13 and 18 years (mean value 15.03; SD = 1.51) referred to acute inpatient treatment for suicidal or impulsive or aggressive behavior.

Adolescents with the origin in the following countries were included: Germany (74%), Syria (10%), Afghanistan (4%), Morocco (1%), Iran (1%), Nigeria (1%), Poland (1%) Pakistan (1%), Gambia (1%), Somalia (1%), USA (1%).

The study inclusion procedure consists of a session with a child and adolescent psychiatrist to evaluate admissibility regarding indication for acute inpatient treatment and to deliver information about the open-group- intervention as well as informed consent. All patients received a handout with information and worksheets at the beginning of the study. After informed consent, adolescents were included in the treatment group. The FEEL-KJ, SCS-13, PSS-10, and SDQ were applied before and after treatment.

Inclusion criteria:

• Age 13–18
• Mental crisis presentation for symptoms of emotional dysregulation
• Inpatient referral for non-suicidal self-harming behavior, aggressive/impulsive behavior, emotional crises
• Voluntary participation in the group program

Exclusion criteria:

• Diagnoses of schizophrenic or affective psychosis

The inclusion criteria in this study are based on symptoms of emotion dysregulation, emotional crisis, perceived stress and severe mental health problems. Inclusion criteria were based on admission in crisis for dysregulated emotions and severe mental distress, not a specific diagnosis. Consent and voluntary participation were basic requirements. Although diagnoses (ICD-10) were documented and are presented below, they were not a prerequisite for participating in the study. On the other hand, our exclusion criteria were based on the diagnosis of schizophrenia or severe affective psychoses.

The diagnostic spectrum of the final sample (see Table 1) consists of a complex combination of response to severe stress and Posttraumatic Stress Disorder (PTSD), adjustment disorder, depressive disorder and borderline personality disorder. Confirmation of these ICD-10-diagnoses follows a clinical routine workflow with standardized diagnostic procedures specific for the disorder in question.

**Table 1:** Main diagnoses—international statistical classification of diseases (ICD-10).

Diagnoses	ICD-10	Frequency	Percent
Mental and behavioural disorders due to psychoactive substance use: harmful use	F19.1	2	1%
Mild depressive episode	F32.0	1	1%
Moderate depressive episode	F32.1	24	15%
Recurrent depressive disorder, current episode moderate	F33.1	1	1%
Social phobias	F40.1	4	3%
Specific (isolated) phobias	F40.2	1	1%
Mixed anxiety and depressive disorders	F41.2	1	1%
Predominantly obsessional thoughts or ruminations	F42.1	1	1%
Acute stress reaction	F43.0	6	4%
Post-traumatic stress disorder	F43.1	55	35%
Adjustment disorders	F43.2	32	21%
Anorexia nervosa, active type	F50.01	1	1%
Bulimia nervosa	F50.2	1	1%
Dissociative convulsions	F44.5	1	1%
Emotionally unstable personality disorder	F60.31	10	6%
Other habit and impulsive disorders	F63.8	1	1%
Disturbance of activity and attention	F90.0	3	2%
Depressive conduct disorders	F92.0	3	2%
Other mixed disorders of conduct and emotions	F92.8	3	2%
Social anxiety disorders of childhood	F93.2	2	1%
Other childhood emotional disorders	F93.8	1	1%
Disinhibited attachment disorder of childhood	F94.2	1	1%

Adolescents were asked to complete the following questionnaires using a PC in a quiet setting accompanied by a therapist in case of comprehensive issues regarding the material or other questions.

### 2.2. Instruments

#### 2.2.1. Questionnaire for the assessment of emotional regulation in children and adolescents (FEEL-KJ)

The FEEL-KJ questionnaire (37, 38) quantifies 15 strategies for emotion regulation and regulation of the specific emotions (all of which are multi-dimensional and specific to a certain emotion): anxiety, sadness, and anger. In two secondary scales, this instrument identifies seven adaptive and five maladaptive emotion regulation strategies. Adaptive strategies include the following sub-scales: problem-oriented acting, distraction, mood improvement, acceptance, forgetting, cognitive problem solving, dealing with anger, anxiety, and grief. Maladaptive strategies include the sub-scales: giving up, aggressive behavior, self-devaluation, withdrawal, perseveration as well as dealing with anger, anxiety and grief. The additional scales are composed of social support, expression, and emotional control.

Internal consistency of the 15 scales ranges between *α* = 0.69 and *α* = 0.91. Secondary scales show a consistency of *α* = 0.93 (adaptive strategies) and *α* = 0.82 (maladaptive strategies). Retest-Reliability (6-week-stability) ranges between *r* = 0.62 and *r* = 0.81, and for the secondary scales between *r* = 0.81 (adaptive strategies) and *r* = 0.73 (maladaptive strategies). The secondary scale, called “adaptive scales” for specific emotions shows a very good internal consistency *α* = 0.88 for sadness, *α* = 0.83 for anxiety, and *α* = 0.83 for anger. The maladaptive scale shows internal consistency for anxiety *α* = 0.59, for sadness *α* = 0.59 and for anger *α* = 0.58.

Furthermore, “additional scales” of the FEEL-KJ provide additional data on the strategies, “expression,” “social support,” and “emotion control,” which are not covered in the two secondary scales. In addition, psychosocial skills and resources are included in this instrument (37).

#### 2.2.2. Self control scale (SCS-13)

The self-control scale depicts the own self-control capacity by means of a five-fold step into the scale using 13 items. High values indicate strongly perceived self-control and low values correspond to lower self-control. SCS was highly reliable: Cronbach's Alpha:.83–.85 and retest-reliability: .87 (32). The German version has been published by Bertrams and Dickhäuser, 2009. On a five-point Likert scale this questionnaire assesses a total score of perceived self-control (33).

#### 2.2.3. Perceived stress scale (PSS-10)

The PSS is a widely and well-established self-report scale for measuring psychological stress. The original tool is a 14-item scale (PSS-14) (38) that was shortened to 10 items (PSS-10) using factor analysis (39), which is used in this study. Participants report the degree to which situations in one's life have been unpredictable, uncontrollable and overloaded in the past month on a five-point Likert scale. The german version shows a good internal consistency (Cronbach's *α* .77) and construct validity. It is associated with depression, anxiety and reduced life satisfaction (40). The total score is calculated from the sum of the items on the helplessness scale and the sum of the inverted items on the self-efficacy scale.

#### 2.2.4. Strenghts and difficulties questionnaire (SDQ)

The SDQ is a screening instrument for mental health problems in children and teenagers. It addresses the positive and negative behavioral attributes on a 25-item scale in a five-factor structure: hyperactivity/inattention, conduct problems, prosocial behavior, peer problems and emotional symptoms. The assessment generates scores for clinically relevant aspects (30).

In a german validation study the five-factor structure was confirmed by exploratory and confirmatory factor analyses. The Total Difficulties Scale showed a satisfactory internal consistency and reliability (Cronbach's *α* .77, retest .66). Reliability of the five scales was low (Cronbach's *α* .55−.77, retest.58−.67) (31).

#### 2.2.5. Experimental intervention

The low-threshold program START (Stress-Traumasymptoms-Arousal-Regulation-Treatment) was developed for adolescents who experience severe stress and emotion dysregulation (29, 41).

The intervention uses treatment strategies from DBT, Tf-CBT and takes inspiration from EMDR into account in active mindfulness exercises. START was manualized for standardized implementation and application. A workbook for adolescents with simple and short exercises that are easy to learn and are demonstrated using pictures. It contains worksheets in English, Italian, Arabic, Dari, and German for all sessions of the treatment program. Since the program is multilingual, it is also suitable for participants with different cultural backgrounds, as initial data has shown (42).

Playful multimedia didactics encourage interest in the START exercises. When developing the program, the feedback from adolescent patients on each exercise was taken into account. In addition, many colored images and illustrations contain the exercises for each module and session. The exercises took into account that heavily stressed adolescents often have a reduced attention span, thus requirements for language, cognitive, and attention span are very low. It was important to the authors that all START participants should be able to successfully complete exercises, enjoy active participation and have a direct sense of achievement. Among other things, resilience should be strengthened. The course of the sessions follows a clearly structured session guide. The intervention was designed as a short-term intervention for five weeks and includes five modules in total. Each module consists of two group sessions. A group session lasts 70 min. In this study, six participants take part in the group setting. Participants were assigned to the groups according to the order of admission. Thereby the groups were randomly mixed with regard to ethnicty and gender. The START program is explicitly designed as a short-term intervention for emotional stabilization and explicitly not as reappraisal or exposure-based psychotherapy. Ultimately, the aim is to promote stress reduction and emotional stabilization. With the help of START, skills on the topics of mindfulness, stress regulation, self-efficacy, emotion awareness, and regulation are taught. START-skills are “abilities” that can help to reduce stress, “manage” crises, and regulate emotions in the short and long term. Another benefit of the program is its design to reduce the barrier to treatment with minimal language dependence. START is intended to be easily implemented, even for participants with low socio-economic backgrounds and low educational levels.

The group is led by two trainers, a therapist (psychotherapist or physician), and a nurse, both of whom received START training via a 4-h START training workshop and START manual in German. The START short-time intervention is easy to establish in inpatient settings.

### 2.3. Data analysis and statistics

All of the analyses were performed with IBM Statistics SPSS, version 27.0. A dependent *t*-test was applied for comparison of pre- vs. post-treatment raw scores and t-values. The hypothesis posits that the 5-week

START intervention shows significant differences as indicated in mean *t*-scores before and after treatment. The focus is emotion regulation measured with FEEL-KJ, perceived stress (PSS-10), self-control (SCS-13), mental impairment (SDQ). The mean value comparison of all pre- and post-measurements was performed based on normalized *t*-values, considering the prerequisites by means of a t-test for dependent samples. The hypothesis postulates that the 5-week START-treatment course will show an effect on emotion regulation (FEEL-KJ), mental health problems and disorders (SDQ), psychological perceived stress (PSS-10) and self-control (SCS-13), as indicated by a significant difference in the mean *t*-values pre- and posttreatment. The mean values were compared with a dependent *t*-test for paired samples. For this trial, in order to assess feasibility, alpha level was adjusted by Bonferroni-Holm method.

While most procedures associate a higher value with a higher symptom score, the FEEL-KJ must be interpreted based on the t-distribution. A value between 25 and 50 is considered as an average for all scales. For adaptive strategies, a lower value is considered to be worse while for maladaptive strategies, a higher value is considered less desirable.

For all scales in the PSS-10 and SDQ a higher score is associated with a higher symptom score. Vice Versa in the SCS-13, a higher value is associated with a higher self-control.

A per-protocol analysis was conducted: Participants who answered all questions of the screenings used and who completed the post-screening were included in the evaluation of the respective questionnaire. Participants with missing information in any questionnaire were excluded from the analysis of the respective questionnaire. Therefore, the amount of participants can vary in numbers, depending on the amount of missing values for different instruments.

## 3. Results

### 3.1. Main diagnoses—international statistical classification of diseases (ICD-10)

The main child psychiatric diagnoses of the participants are shown in Table 1. The most common diagnoses are PTSD) and adjustment disorder. The diagnoses were recorded with instruments of clinical diagnostics, anamnestic information, and specialized diagnostic instruments such as Child and Adolescent Trauma Screen (CATS) and Child Post-traumatic Cognitions Inventory (CPTCI-25).

Based on the objectives of START-Program the following questions and hypotheses are of interest for the present study: The central question was whether the five-week START program can influence emotion regulation. The pre/post survey was carried out with the FEEL-KJ. Furthermore, it was of interest whether the perceived stress changed, for which the PSS-10 was used. The SCS-13 questionnaire aimed to measure a potential change in self-control after the intervention phase. Changes in both strengths and difficulties were also evaluated. Although no diagnoses were specified for inclusion in the study, the main diagnoses were determined as covariates using anamnesis and clinical procedures such as Child and Adolescent Trauma Screen (CATS) and Child Post-Traumatic Cognitions Inventory (CPTCI).

### 3.2. Questionnaire for the assessment of the emotional regulation in children and adolescents (FEEL-KJ)

#### 3.2.1. FEEL-KJ: adaptive scales

Adaptive strategies include a total scale of adaptive strategies and the following sub-scales: problem-oriented acting, distraction, mood improvement, acceptance, forgetting, cognitive problem solving, dealing with anger, anxiety and grief.

Significant improvements in adaptive strategies can be observed after the START intervention. The total of adaptive strategies *(d = *0.25*, p = *.001*)* was significantly increased. The significant improvements are also shown on the subscales as follows: problem-oriented acting *(d = *0.28*, p = *.001*),* distraction *(d *= .30*, p *< .001*)*, mood improvement *(d *= .004*, p *= 0.24*)*, acceptance (*d *= .24, *p *= .006) cognitive problem solving *(d = *.30*, p *< .001*)*.

**Figure 1:**
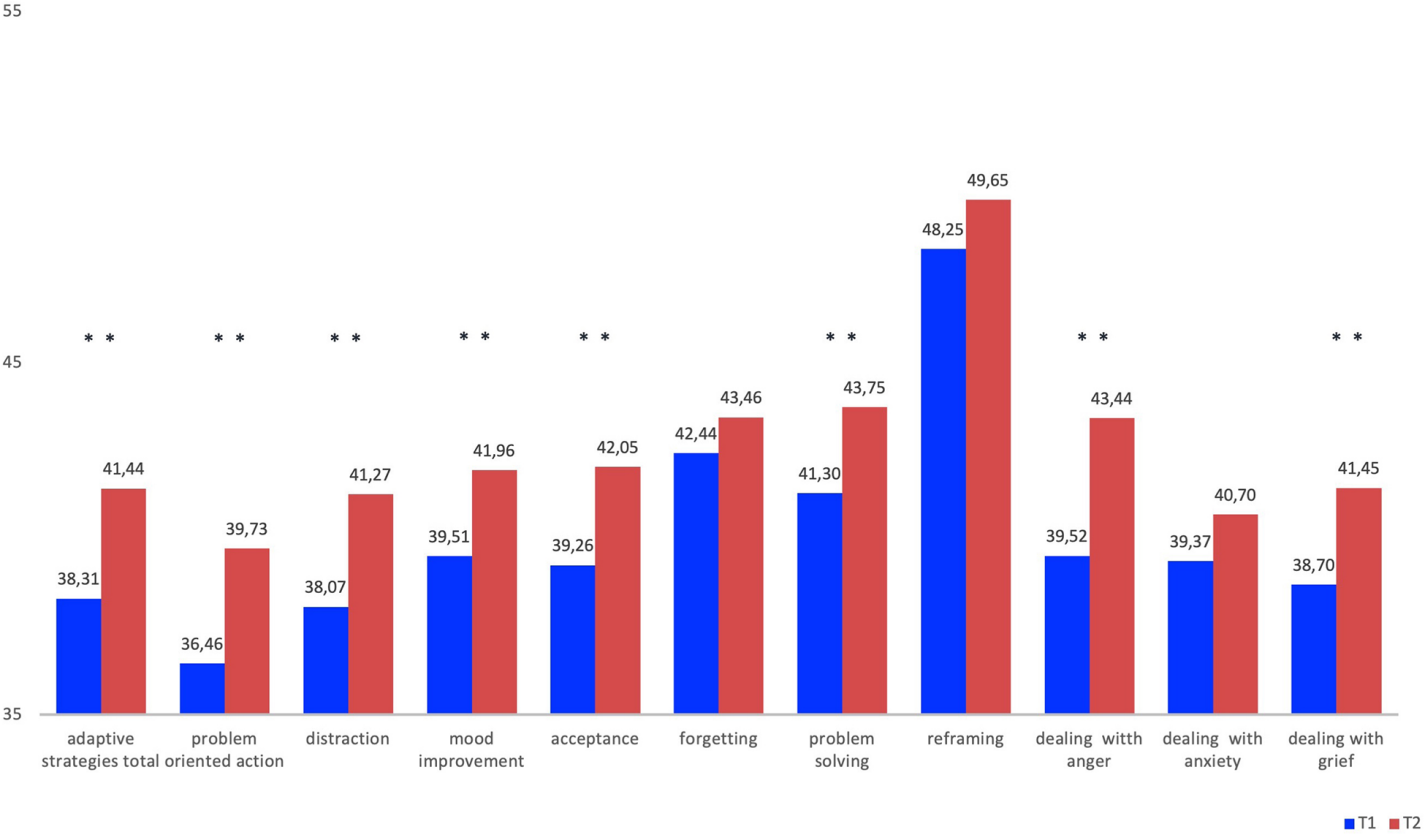
FEEL-KJ adaptive scales.

The subscales forgetting *(d = *.009*, p = *.273*)* and reframing *(d *= .011*, p *= .160*)* show no significant improvement. The adaptive strategies for the specific emotions of dealing with anger *(d = *.036*, p = *.001*) and* dealing with grief *(d = *.28*, p = *.001*)* are significantly improved after the START intervention. Strategies of dealing with anxiety *(d = *.012*, p = *.152*)* show no significant changes. Detailed information is given in Table 2 and Figure 1.

**Table 2:** FEEL-KJ adaptive scales.

FEEL-KJ	Mean T1 (±SD)	Mean T2 (±SD)	Mean difference (±SD)	*t*	*n*	*p*	Cohens d
Adaptive strategies total	38.31 (12.17)	41.44 (13.04)	−3.12 (11.56)	−3.34	154	.001	0.25
Problem oriented action	36.46 (11.08)	39.73 (12.59)	−3.27 (12.20)	−3.31	154	.001	0.28
Distraction	38.07 (10.32)	41.27 (11.28)	−3.20 (10.72)	−3.69	154	<.001	0.30
Mood improvement	39.51 (9.88)	41.96 (10.76)	−2.45 (10.29)	−2.95	154	.004	0.24
Acceptance	39.26 (10.62)	42.05 (12.50)	−2.79 (12.29)	−2.81	154	.006	0.24
Forgetting	42.44 (11.66)	43.46 (34.47)	−3.64 (34.79)	−1.10	154	.273	0.09
Problem solving	41.30 (12.30)	43.75 (12.09)	−2.44 (11.12)	−2.72	154	.007	0.20
Reframing	48.25 (12.12)	49.65 (12.35)	−1.40 12.24)	−1.41	154	.160	0.11
Dealing with anger	39.52 (10.40)	43.44 (11.36)	−3.92 (10.95)	−4.42	154	<.001	0.36
Dealing with anxiety	39.37 (11.13)	40.70 (11.66)	−1.33 (11.44)	−1.44	154	.152	0.12
Dealing with grief	38.70 (10.64)	41.86 (11.97)	−3.16 (11.98)	−3.62	154	.001	0.28

#### 3.2.2. FEEL-KJ: maladaptive strategies

The results of the total scale of maladaptive regulation strategies *(d *= −0.24*, p *= .001) decrease after START intervention and changes significantly*.* Also, the sub-scales giving up *(d *= −0.31*, p *= .001*),* withdrawal *(d *= .004*, p *= 0.24*)* and the emotions dealing with grief *(d *= −0.22*, p *= .001 and dealing with anxiety *(d *= −0.22*, p *= .004*)* show significant changes. In contrast, there was no significant decrease in the maladaptive strategies in particular with regard to the strategies aggressive behavior *(d *= −0.02*, p *= .762*),* perseveration *(d *= −0.09, *p *= .234*)* and self-devaluation *(d *= −0.09*, p *= .395*).* An improvement is also seen for the maladaptive strategy of the specific emotion dealing with anger *(d *= −0.22*, p *= .003*).* However, the maladaptive strategies for this study are not presented in tables and figures for dissatisfactory reliability characteristics of this subscale of the FEEL-KJ.

#### 3.2.3. FEEL-KJ: additional scales

**Figure 2:**
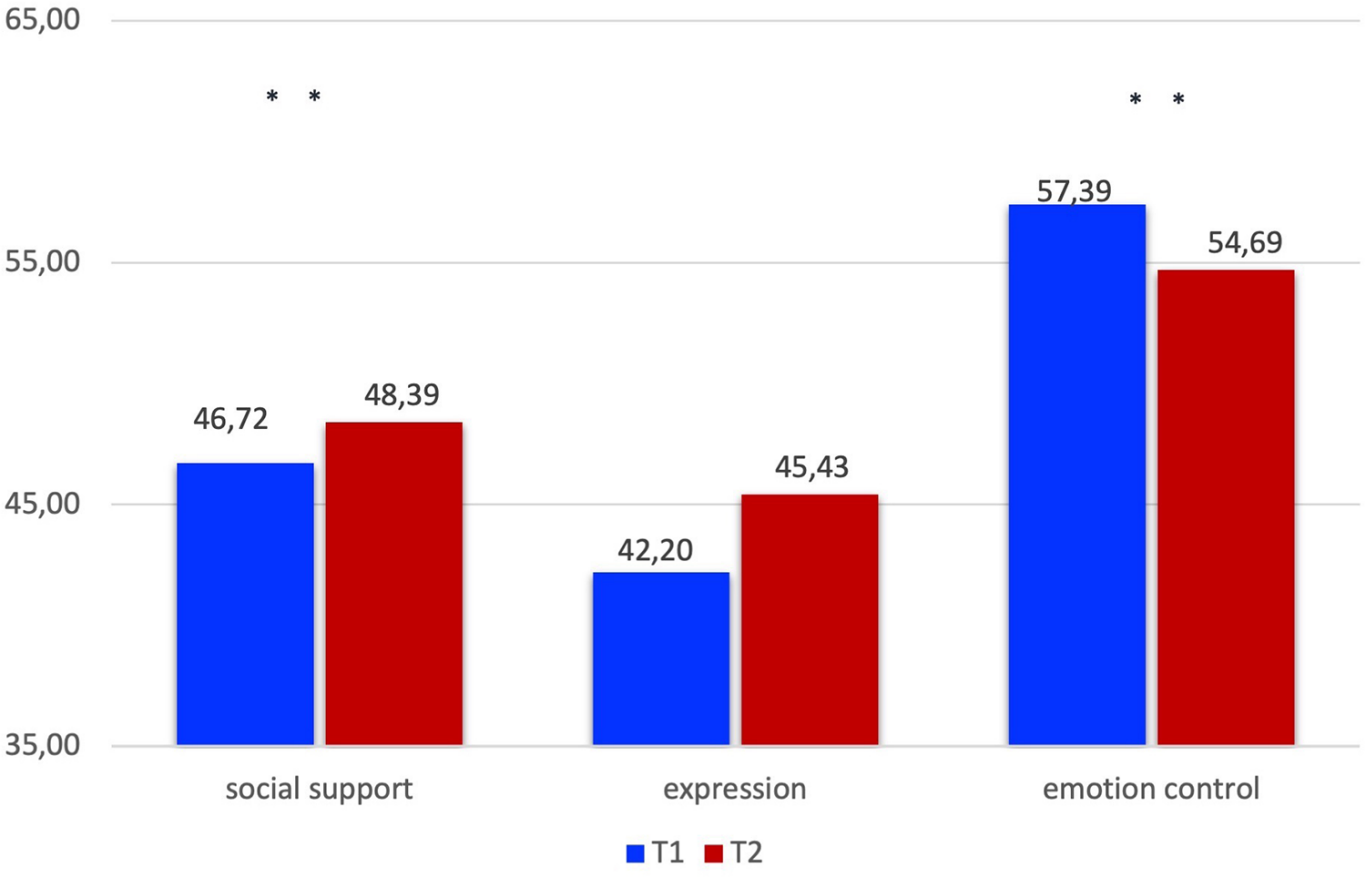
FEEL-KJ additional scales.

Regarding the additional strategies (see Table 3, Figure 2), a significant improvement was shown on social support *(d *= 0.31*, p* < .001*)* and a significant reduction of emotional control *(d *= −0.23*, p *= .002*)*. With regard to the control of emotions, there was a reduction within the standard. The emotion control was above average before the START intervention. The decrease from the above-average value is to be regarded as an increase in the perception of emotions to understand. No significant difference was observed in emotion expression *(d *= 0.14, *p *= .170*)*.

**Table 3:** FEEL-KJ additional scales.

FEEL-KJ	Mean T1 (±SD)	Mean T2 (±SD)	Mean difference (±SD)	*t*	*n*	*p*	Cohens d
Expression	46.72 (11.58)	48.39 (11.75)	−1.67 (8.53)	−2.42	154	.170	0.14
Social support	42.20 (10.20)	45.43 (10.45)	−3.23 (8.52)	−4.69	154	<.001	0.31
Emotion control	57.39 (11.83)	54.69 (11.93)	2.70 (10.85)	3.08	154	.002	−0.23

### 3.3. Perceived stress scale (PSS-10)

The total score is calculated from the sum of the items on the helplessness scale and the sum of the inverted items on the self-efficacy scales (see Table 4). The helplessness scale (H) is the sum of items 1, 2, 3, 6, 9, 10. For evaluating the self-efficacy scale (S) the sum must be determined by the items 4, 5, 7, 8. To calculate the total score, items 4, 5, 7 and 8 of the self-efficacy scale must be inverted. Higher values indicate an increased stress level.

**Table 4:** Perceived stress scale (PSS-10); self control scale (SCS-13); strengths and difficulties questionnaire (SDQ).

PSS-10	Mean T1 (±SD)	Mean T2 (±SD)	Mean difference (±SD)	*t*	*n*	*p*	Cohens d
	3.51 (0.69)	3.27 (0.74)	0.24 (0.71)	4.04	147	<.001	−0.34
SCS-13							
	2.74 (0.70)	2.90 (0.83)	−0.16 (0.56)	−3.54	156	.001	0.21
SDQ							
Total	19.28 (5.74)	18.19 (6.85)	1.09 (5.65)	2.40	156	.018	−0.17
Emotional problems	6.85 (2.59)	6.44 (2.93)	0.41 (2.34)	2.20	156	.029	−0.15
Behavior problems	3.11 (2.02)	2.79 (1.98)	0.32 (2.05)	1.96	156	.052	−0.16
Hyperactivity	4.93 (2.32)	4.61 (2.59)	0.32 (2.04)	1.93	156	.056	−0.13
Peer problems	4.45 (2.05)	4.54 (2.84)	−0.09 (2.76)	−0.41	156	.684	0.04
Prosocial behavior	8.04 (2.29)	8.08 (2.51)	−0.04 (2.32)	−0.21	156	.835	0.02

Our assessment revealed significantly lower scores post-intervention in psychological stress (PSS-10) *(d *= −0.34*, p *< .001*)*.

### 3.4. Self control scale (SCS-13)

The Self Control Score (SCS-13) increased significantly after the intervention (*d* = 0.21, *p *= .001), values and *t*-Scores are shown in table (see Table 4).

### 3.5. Strengths and difficulties questionnaire (SDQ)

On the one hand, the SDQ questionnaire records mental stress or difficulties in relation to mental problems and behavioral aspects. On the other hand, the positive characteristics, skills, and resources are also recorded. It is particularly helpful in the questionnaire to consider the influence on the environment and the functional level of the children and young people.

The scores (SDQ)show a significant decrease after the intervention (see Table 4) in the range and scale emotional problems (*d *= −0.17, *p *= .018). There are no significant changes in the pre/post comparison for the scales behavior problems (d = −0.16*, p *= .052)*,* hyperactivity (*d = −0.13, p *= .056)*,* peer problems, (*d *= 0.04*, p *= .684) and prosocial behavior (*d *= 0.02*, p *= .835).

## 4. Discussion

The intervention evaluated in this study was designed to offer a first aid for crisis management and emotional stabilization (31, 43) to emotionally dysregulated adolescents in acute need of inpatient psychiatric treatment for suicidal behavior, and to patients with (auto)-aggressive behavior, even with short attention span, low motivation and/or limited cognitive abilities. The results presented here could support the applicability and efficacy of START with an additional advantage of integration and strengthening of resilience in several populations at risk. While some of the effects reported here are of greater significance, some scales have shown only very marginal improvement, specifically SDQ emotional problems, SCS13 and FEEL-KJ adaptive scales, with a group difference of less than 0.3 standard deviations. However the direction of the effects is very satisfactory, regarding the fact that patients could also deteriorate in acute settings as has clinically been. Reaching sufficient power for proving clinical benefit is a frequent challenge in therapeutic interventions (44). According to the data presented here**,** our program achieves fast behavioral and emotional stabilization for adolescents with chronic and/or acute stress. Therefore, it might be a highly useful and cost-saving tool for patients with acute emotion dysregulation because the length of treatment can be decreased and hospital re-admissions and subsequent disintegration might be avoided, thus saving mental health costs. The return of investment in human capital in general is highest if invested early in life, as cerebral plasticity is highest and learning effects can be easier achieved and permanently used, thereby achieving a large impact with smaller interventions. Cost saving might extend to many life aspects, from children's lifespans to issues such as mental health costs, social costs for unemployment, bad parenting skills or other consequences or poor emotion regulation.

This includes tools to become aware of emotional tension and skills to reduce it. The program thereby aims at reducing behaviors including harming of self- and others as well as impulsive behavior. The program is manualized and characterized methodologically. The applied intervention is designed to have a low threshold and to be very playful in terms of the addressed target population with symptom clusters of severe emotional distress, frequently being not motivated to take part in any kind of low-stimulus -psychotherapy.

The short duration of the treatment program was an important aspect: In adolescents, motivation to seek treatment, as well as adherence and compliance are often limited. Therefore, it seemed necessary to design a program not requiring a commitment for longer than 5 weeks, as this might specifically be helpful for this unstable group of young patients.

The authors of the program (29) tried to fill a therapeutic gap (7) across diagnoses by providing a highly structured manual, aiming at adolescents with a wide range of psychiatric diagnoses and emotional dysregulation. Due to the need for standardized settings, our study presents data gathered in a clinical in-patient setting immediately before and after treatment. We could show in our study that the use of adaptive regulation strategies for acute crisis management and regulation of emotions was significantly improved by our program. Furthermore, our data indicates that perceived stress as well as general psychopathology assessed by SDQ were significantly reduced, pointing to the fact that improving recognition and regulation of emotions plays a crucial role in overall well-being and mental health. Self-control could influence a positive development, which is helpful for integration in social contexts. Self-control (34) is an ability or skill for controlling impulses and modifying inappropriate emotions and thoughts. The ability to effectively regulate emotions allows individuals to cope with stress, navigate through difficult situations, and maintain healthy relationships. Correlated with emotional dysregulation are psychiatric symptoms such as anxiety, depression, impulsivity, and irritability, which can negatively impact their daily lives and overall functioning. Improving emotion regulation can lead to improved mood, better stress management, and a higher quality of life. Early intervention and treatment can help prevent the development of more severe mental health conditions and improve the child's overall quality of life (43). Child psychiatry professionals can work with families to develop individualized treatment plans that address the unique needs and challenges of each child with emotion dysregulation (24). Future studies with larger sample sizes should assess whether there are effects of gender or ethinicity on feasibility and effectivenes of START in order to provide specific adaptations potentally becoming necessary by potentially established differences.

Because a high arousal and emotional tension such as in a child psychiatric population with acute in-patient- presentation is often combined with substance abuse problems (45) the skills are designed to improve these capacities, leading to a more successful life span as described above.

A presumed improvement of self-control (34) and self-confidence is potentially accompanied by the reduction of non-adaptive behavior with subsequent negative labeling by the immediate environment. Future studies should assess aspects of self- worth and identity in a randomized control design regarding the treatment program.

### 4.1. Limitations

Limitations of our study are the lack of a treatment—as usual—control group. This shall be addressed in future studies, after this assessment has shown promising results from this acute treatment program. Therefore, the proven feasibility and large number of benefitting adolescents give rise for the construction of a randomized control trial.

A multicenter consortium is required to provide sufficient clinical data in a randomized control trial and is underway (45). This ongoing RCT might contribute to close the gap between emotion dysregulation in minors and the lack of clinical tools and evaluation in a joint effort intervention program.

### 4.2. Clinical impact

An improvement in emotion regulation and a decrease in mental health problems in adolescents with presentation of acute emotional instability can improve the social and emotional as well as academic development in this vulnerable group of adolescent patients. Our data shows the beneficial impact on emotion regulation. This points to a positive clinical and societal impact as severe emotional dysregulation can be the primary cause for admission to psychiatric wards and pose a significant hurdle for the successful intervention for core symptoms of the primary disorder. In consequence, length of stay on emergency/closed wards is increased, posing a significant burden on patients and their families, but also on the health care system. An improvement in emotion regulation can improve the social and emotional as well as academic development in children and adolescents. This seems to be of increasing importance since early emotion dysregulation in children and adolescents predicts a range of psychiatric, general medical and social problems in adolescence and young adulthood, emphasizing its public health significance. Adolescents with low emotion regulation skills tend to experience significant social impairments e.g., relationship difficulties with parents, siblings, and teachers, school suspension, service use (mental health and general medical), drug abuse, harm to self and others and poverty. An improvement in emotion regulation as was shown by our novel, low threshold intervention, can improve the social and emotional as well as academic development in young people.

Further novel aspects of our program are potential links of the clinical trials to preclinical approaches, which has not been realized before, like in a second step introducing and evaluating START in school and youth welfare settings. Therefore, apart from the evaluation setting presented here, a broad application of the intervention beyond clinical settings seems possible. Therefore, on another note it will be explored whether this low-threshold program might be extended to preclinical approaches, which has not been realized before, e.g., introducing START in school settings or the youth welfare system. With increasing mental health needs, these approaches seem to be necessary and helpful in reducing barriers to treatment, such as limited availability, which are detrimental specifically in the young population.

## Data Availability

The original contributions presented in the study are included in the article, further inquiries can be directed to the corresponding authors.

## References

[1] De Berardis D Fornaro M Orsolini L Ventriglio A Vellante F Di Giannantonio M. Emotional dysregulation in adolescents: implications for the development of severe psychiatric disorders, substance abuse, and suicidal ideation and behaviors. Brain Sci. (2020) 10(9):591. 10.3390/brainsci10090591 32858969 PMC7565002

[2] Gross JJ Jazaieri H. Emotion, emotion regulation, and psychopathology: an affective science perspective. Clin Psychol Sci. (2014) 2(4):387–401. 10.1177/2167702614536164

[3] Gross JJ. The emerging field of emotion regulation: an integrative review. Rev Gen Psychol. (1998) 2(3):271–99. 10.1037/1089-2680.2.3.271

[4] Gross JJ. Handbook of emotion regulation. 2nd ed. New York: Database: APA PsycInfo: The Guilford Press (2014).

[5] Aldao A Nolen-Hoeksema S Schweizer S. Emotion-regulation strategies across psychopathology: a meta-analytic review. Clin Psychol Rev. (2010) 30(2):217–37. 10.1016/j.cpr.2009.11.004 20015584

[6] Felliti V Andra R Nordenberg D Williamson D Spitz A Edwards V Relationship of childhood abuse and household dysfunction to many of the leading causes of death in adults. The adverse childhood experiences (ACE) study. Am J Prev Med. (1998) 14(4):245–58. 10.1016/S0749-3797(98)00017-8 9635069

[7] Dixius A Möhler E. Feasibility and effectiveness of a new short-term psychotherapy concept for adolescents with emotional dysregulation. Front Psychiatry. (2021) 11:585250. 10.3389/fpsyt.2020.585250 33551862 PMC7858646

[8] Cohen J Mannarino A Deblinger E. Traumafokussierte kognitive Verhaltenstherapie bei Kindern und Jugendlichen. 1st ed. Heidelberg: Springer Verlag (2009).

[9] Rosner R Steil R. Ratgeber posttraumatische belastungsstörung informationen für betroffene, eltern, lehrer und erzieher. 1. Auflage. Göttingen: Hogrefe Verlag GmbH & Co. KG (2009).

[10] Steil R Straube ER. Posttraumatische belastungsstörung bei kindern und jugendlichen. Z Für Klin Psychol Psychother. (2002) 31(1):1–13. 10.1026/0084-5345.31.1.1

[11] Klonsky E. The functions of deliberate self-injury: a review of the evidence. Clin Psychol Rev. (2007) 27(2):226–39. 10.1016/j.cpr.2006.08.002 17014942

[12] In-Albon T Plener P Brunner R Kaess M. Ratgeber selbstverletzendes verhalten. 1st ed. Göttingen: Hogrefe Verlag GmbH & Co. KG (2015).

[13] Plener P Schumacher T Munz L Grosschwitz R. The longitudinal course of non-suicidal self-injury and deliberate self-harm: a systematic review of the literature. Bord Pers Discord Emot Dysregul. (2015) 2:2. 10.1186/s40479-014-0024-3 PMC4579518 26401305

[14] Schmid M Fegert JM Petermann F. Traumaentwicklungsstörung: pro und contra. Kindh Entwickl. (2010) 19(1):47–63. 10.1026/0942-5403/a000008

[15] Krüsmann M Müller-Cyran A. Trauma und frühe interventionen: möglichkeiten und grenzen von krisenintervention und notfallpsychologie. Stuttgart: Pfeiffer bei Klett-Cotta (2005).

[16] Axelson D Findling RL Fristad MA Kowatch RA Youngstrom EA Horwitz SM Examining the proposed disruptive mood dysregulation disorder diagnosis in children in the longitudinal assessment of manic symptoms study. J Clin Psychiatry. (2012) 73(10):1342–50. 10.4088/JCP.12m07674 23140653 PMC3581334

[17] Brunner R Parzer P Haffner J Steen R Roos J Klett M Prevalence and psychological correlates of occasional and repetitive deliberate self-harm in adolescents. Arch Pediatr Adolesc Med. (2007) 161(7):641–9. 10.1001/archpedi.161.7.641 17606826

[18] Johnson JG Cohen P Brown J Smailes EM Bernstein DP. Childhood maltreatment increases risk for personality disorders during early adulthood. Arch Gen Psychiatry. (1999) 56(7):600. 10.1001/archpsyc.56.7.600 10401504

[19] Sansone R Gaither G Songer D. The relationships among childhood abuse, borderline personality, and self-harm behavior in psychiatric inpatients. Violence Vict. (2002) 17(1):49–55. 10.1891/vivi.17.1.49.33636 11991156

[20] Möhler E Resch F. Maternal salivary cortisol in pregnancy and pre-, peri- and postnatal medical complications. J Preg Child Health. (2018) 2018(01). 10.29011/JPCH-101.100001

[21] Herpertz SC Dietrich TM Wenning B Krings T Erberich SG Willmes K Evidence of abnormal amygdala functioning in borderline personality disorder: a functional MRI study. Biol Psychiatry. (2001) 50(4):292–8. 10.1016/S0006-3223(01)01075-7 11522264

[22] Bohus M. Borderline-Störung. Vol. 14. 2nd ed. Göttingen: Hogrefe Verlag GmbH & Co. KG (2019).

[23] Sharp C Pane H Ha C Venta A Patel AB Sturek J Theory of mind and emotion regulation difficulties in adolescents with borderline traits. J Am Acad Child Adolesc Psychiatry. (2011) 50(6):563–573.e1. 10.1016/j.jaac.2011.01.017 21621140

[24] Chanen AM McCutcheon LK Jovev M Jackson HJ McGorry PD. Prevention and early intervention for borderline personality disorder. Med J Aust. (2007) 187(S7):S18–S21. 10.5694/j.1326-5377.2007.tb01330.x 17908019

[25] Fegert J Freyberger H. Posttraumatische Belastungsstörungen (PTBS) und Traumafolgestörungen in der Adoleszenz. Fortschritte Neurol Psychiatr. (2019) 87:638–41. 10.1055/a-1016-3316 31756746

[26] Paulus FW Ohmann S Möhler E Plener P Popow C. Emotional dysregulation in children and adolescents with psychiatric disorders. A narrative review. Front Psychiatry. (2021) 12:628252. 10.3389/fpsyt.2021.628252 34759846 PMC8573252

[27] Nezlek JB Kuppens P. Regulating positive and negative emotions in daily life. J Pers. (2008) 76(3):561–80. 10.1111/j.1467-6494.2008.00496.x 18399953

[28] Möhler E Resch F. Early life stress. Prax Kinderpsychol Kinderpsychiatr. (2019) 68(7):575–91. 10.13109/prkk.2019.68.7.575 31711395

[29] Dixius A Möhler E. *START—Stress-Traumasymptoms-Arousal-Regulation Treatment. Manual zur Erststabilisierung und Arousal-Modulation für stark belastete Kinder und Jugendliche und minderjährige Flüchtlinge*. Saarbrücken. Available at: startyourway.de 10.13109/prkk.2017.66.4.277 28393646

[30] Goodman R. The strengths and difficulties questionnaire: a research note. J Child Psychol Psychiatry. (1997) 38(5):581–6. 10.1111/j.1469-7610.1997.tb01545.x 9255702

[31] Lohbeck A Schultheiß J Petermann F Petermann U. Die deutsche selbstbeurteilungs- version des strengths and difficulties questionnaire (SDQ-deu-S). Diagnostica. (2015) 61(4):222–35. 10.1026/0012-1924/a000153

[32] Tagney J Baumeister R Boone A. High self-control predicts good adjustment, less pathology, better grades, and interpersonal success. J Pers. (2004) 72(2):271–324. 10.1111/j.0022-3506.2004.00263.x 15016066

[33] Bertrams A Dickhäuser A. Messung dispositioneller Selbstkontroll-Kapazität: Eine deutsche Adaptation der Kurzform der Self-Control Scale (SCS-K-D). Diagnostica. (2009) 55(1):2–10. 10.1026/0012-1924.55.1.2

[34] Smith C Carlson BE. Stress, coping, and resilience in children and youth. Soc Serv Rev. (1997) 71(2):231–56. 10.1086/604249

[35] Felton JW Banducci AN Shadur JM Stadnik R MacPherson L Lejuez CW. The developmental trajectory of perceived stress mediates the relations between distress tolerance and internalizing symptoms among youth. Dev Psychopathol. (2017) 29(4):1391–401. 10.1017/S0954579417000335 28318473 PMC6360527

[36] Gross JJ. Emotion regulation: current Status and future prospects. Psychol Inq. (2015) 26(1):1–26. 10.1080/1047840X.2014.940781

[37] Grob A Smolenski C. Fragebogen zur erhebung der emotionsregulation bei kindern und jugendlichen (FEEL-KJ). 2nd ed. Bern: Huber (2009). Available at: https://books.google.de/books?id=DqeNGwAACAAJ

[38] Cohen S Kamarck T Mermelstein R. A global measure of perceived stress. J Health Soc Behav. (1983) 24(4):385–96. 10.2307/2136404 6668417

[39] Cohen S. Perceived stress in a probability sample of the United States. Soc Psychol Health Sage Publ. (1988):31–67.

[40] Klein E Brähler E Dreier M Reinecke L Müller K Schmutzer G The German version of the perceived stress scale—psychometric characteristics in a representative German community sample. BMC Psychiatry. (2016) 16:1–10. 10.1186/s12888-016-0875-9 27216151 PMC4877813

[41] Dixius A Stevens A Möhler E. A pilot evaluation study of an intercultural treatment program for stabilization and arousal modulation for intensely stressed children and adolescents and Minor refugees, called START (stress-traumasymptoms-arousal-regulation-treatment). ARC J Psychiatry. (2017) 2(2):7–14.

[42] Dixius A Möhler E. Stress-Traumasymptoms-Arousal-Regulation-Treatment (START). Paediatr Paedol. (2018) 53(1):34–8. 10.1007/s00608-018-0585-2

[43] Kaess M Herpertz SC Plener PL Schmahl C. Borderline-Persönlichkeitsstörungen. Z Für Kinder- Jugendpsychiatrie Psychother. (2020) 48(6):1–5. 10.1024/1422-4917/a000700 31755846

[44] Michael T Schanz CG Mattheus HK Issler T Frommberger U Köllner V Do adjuvant interventions improve treatment outcome in adult patients with posttraumatic stress disorder receiving trauma-focused psychotherapy? A systematic review. Eur J Psychotraumatology. (2019) 10(1):1634938. 10.1080/20008198.2019.1634938 PMC6711134 31489131

[45] Sobanski E Hammerle F Dixius A Möhler E Koudela-Hamila S Ebner-Priemer U START Adolescents: study protocol of a randomised controlled trial to investigate the efficacy of a low-threshold group treatment programme in traumatised adolescent refugees. BMJ Open. (2021) 11(12):e057968. 10.1136/bmjopen-2021-057968

[46] Basedow LA Kuitunen-Paul S Roessner V Golub Y. Traumatic events and substance use disorders in adolescents. Front Psychiatry. (2020) 11:559. 10.3389/fpsyt.2020.00559 32625122 PMC7314975

